# Evidence that agricultural use of pesticides selects pyrethroid resistance within *Anopheles gambiae* s.l. populations from cotton growing areas in Burkina Faso, West Africa

**DOI:** 10.1371/journal.pone.0173098

**Published:** 2017-03-02

**Authors:** Aristide Sawdetuo Hien, Dieudonné Diloma Soma, Omer Hema, Bazoma Bayili, Moussa Namountougou, Olivier Gnankiné, Thierry Baldet, Abdoulaye Diabaté, Kounbobr Roch Dabiré

**Affiliations:** 1 IRSS/DRO, Malaria and Tropical Neglected Research Unit, Bobo-Dioulasso, Burkina Faso; 2 Programme Coton/INERA/ Farako-Ba, Bobo-Dioulasso, Burkina Faso; 3 Institute of Rural Development, Université Polytechnique de Bobo-Dioulasso, Burkina Faso; 4 UFR/Life Science, Université Joseph Ki-Zerbo, Ouagadougou, Burkina Faso; 5 CIRAD, Campus de Baillarguet, Montpellier, France; Centro de Pesquisas Rene Rachou, BRAZIL

## Abstract

Many studies have shown the role of agriculture in the selection and spread of resistance of *Anopheles gambiae* s.l. to insecticides. However, no study has directly demonstrated the presence of insecticides in breeding sources as a source of selection for this resistance. It is in this context that we investigated the presence of pesticide residues in breeding habitats and their formal involvement in vector resistance to insecticides in areas of West Africa with intensive farming. This study was carried out from June to November 2013 in Dano, southwest Burkina Faso in areas of conventional (CC) and biological cotton (BC) growing. Water and sediment samples collected from breeding sites located near BC and CC fields were submitted for chromatographic analysis to research and titrate the residual insecticide content found there. Larvae were also collected in these breeding sites and used in toxicity tests to compare their mortality to those of the susceptible strain, *Anopheles gambiae* Kisumu. All tested mosquitoes (living and dead) were analyzed by PCR for species identification and characterization of resistance genes. The toxicity analysis of water from breeding sites showed significantly lower mortality rates in breeding site water from biological cotton (WBC) growing sites compared to that from conventional cotton (WCC) sites respective to both *An*. *gambiae* Kisumu (WBC: 80.75% vs WCC: 92.75%) and a wild-type strain (49.75% vs 66.5%). The allele frequencies L1014F, L1014S *kdr*, and G116S ace -*1*^*R*^ mutations conferring resistance, respectively, to pyrethroids and carbamates / organophosphates were 0.95, 0.4 and 0.12. Deltamethrin and lambda-cyhalothrin were identified in the water samples taken in October/November from mosquitoes breeding in the CC growing area. The concentrations obtained were respectively 0.0147ug/L and 1.49 ug/L to deltamethrin and lambdacyhalothrin. Our results provided evidence by direct analysis (biological and chromatographic tests) of the role of agriculture as a source of selection pressure on vectors to insecticides used in growing areas.

## Introduction

The current control strategy of malaria is mainly based on limiting human-vector contact by the use of insecticide-treated nets (ITNs), although this last decade, indoor residual spraying (IRS) of insecticides has scaled up many African countries [1]. Pyrethroids are the class of insecticides used to impregnate mosquito nets, because they are effective at low doses and exhibit very little toxicity to humans and the environment [2]. In recent years, ITNs have shown good effectiveness in Gambia and Tanzania by reducing malaria-related morbidity and mortality among children aged 0–5 years [3], as well as in a in a stable transmission zone in Ghana [4]. However, the situation has changed significantly in Benin, where the decreased effectiveness of ITNs has recently been observed [5]. Indeed, in Africa, the first case of resistance of *An*. *gambiae* sl to pyrethroids was reported in Ivory Coast [6], and then the phenomenon spread from the Ivory Coast to Burkina Faso [7] a few years later and elsewhere in West Africa [8, 9, 10, 11] with a correlation between resistance and the involvement of the use of the insecticide in agriculture, particularly in the cotton growing regions [12, 13].

Moreover, many studies have demonstrated that mosquito larvae exposed to sub-lethal doses of pollutants, herbicides or pesticides frequently appear more tolerant to insecticides through the induction of their detoxifying system and eventually with other mechanisms, such as resistance genes (*kdr* and ace-*1*^*R*^) [14, 15]. Indeed, in Cameroon *An*. *gambiae* s.l. females were reported to frequently lay their eggs in breeding sites located around agricultural settings, suggesting that larvae may undergo a selection pressure from agricultural pesticides favoring the emergence of resistance [16]. In Khartoum (Sudan), the resistance of *Anopheles arabiensis* to pyrethroids through metabolic resistance was associated with pesticide usage in an intensive agriculture area [17]. More recently, another Tanzanian field study comparing urban, agricultural and low pollution areas pinpointed the elevated resistance level of *An*. *gambiae* s.s. found in proximity of intensive agriculture and identified candidate genes associated with the use of pesticides in agriculture and insecticide resistance [18]. In Burkina Faso, the frequency of resistance genes *(kdr*) was higher in cotton growing areas usually subjected to insecticide treatments than in rural areas where only food crops were grown without insecticides [19]. Indeed, the emergence and distribution of the resistance to insecticides of *Anopheles gambiae* s.l. perfectly overlaps the cotton areas where there is an increase of allelic frequencies of *kdr* mutations L1014F and ace-*1*^*R*^ [20, 21] within the past five years and a very extensive geographic spread of resistance towards new cotton areas previously free of resistance [22]. These examples of agricultural-related resistance threaten the efficacy of insecticide-based vector control (LLIN) tools, particularly in West Africa [5, 22, 23].

In order to restore the efficacy of vector control while waiting for new insecticides that could replace existing tools, a resistance management plan recommended by the WHO has been put in place to improve control [24]. This plan involves a frank collaboration between agriculture and public health domains in the management of the use of insecticides.

Several studies suggest that the selection pressure of insecticide resistance is primarily of agricultural origin, although the contribution of multiple distributions of LLINs to the selection of vector resistance cannot be discounted [25]. However, no direct evidence has yet been shown that supports the direct contamination of pesticides into mosquito breeding sites that would lead to resistance selection. Our study is part of an Ecohealth concept to establish evidence of this relationship, and if so, it could serve as a point of discussion between agriculture and public health entities on this growing problem.

## Materials and methods

### Study area

**Fig 1:**
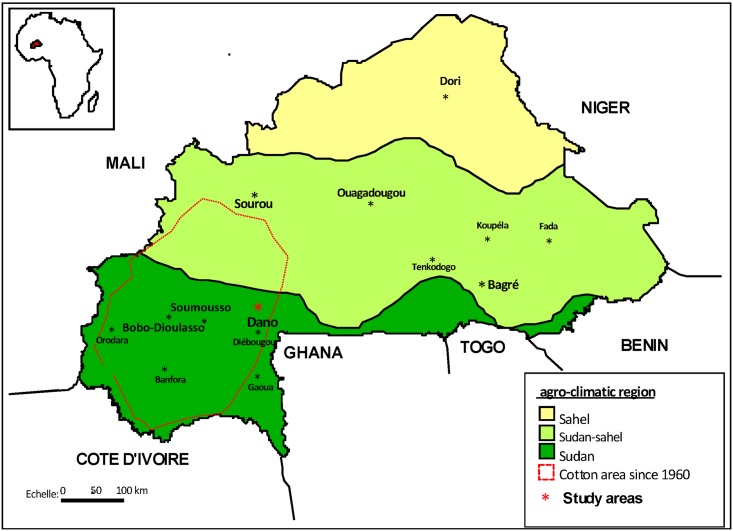
Figure showing the location of the cotton region since 1960 in Burkina Faso and the sampling sites (Dano, Soumousso and Bobo-Dioulasso). The study took place near the town of Dano in two neighboring villages (Kompla and Dobao).

This study was performed in two villages from around the town of Dano, Burkina Faso (Fig 1). The study site was selected due to its easy accessibility and the presence of cotton agriculture that uses both conventional and biological (or organic) growing methods. Conventional and organic cotton are the same crop species, but their growing methods are different. Conventional cotton insecticide applications usually occur in six treatments over fifteen day intervals. These insecticide treatments are applied in rotation and include pyrethroids and organophosphates / carbamates. In contrast, biological cotton is grown using only biological insecticides such as neem extracts.

Dano (11° 08 '26 "N, 3° 03' 50" W) is located southwestern Burkina Faso and situated in woodland savannah. The mean annual rainfall is 1200 mm, which mostly falls from May to October. Malaria transmission is from June to November. *An*. *gambiae* s.l. is the most important malaria vector, followed by *Anopheles funestus*, which is more prevalent towards the end of the rainy season. Previous studies in this locality showed high resistance of *Anopheles gambiae* s.l. and particularly its S form to insecticides, owing to a high frequency (0.971 and 0.898, respectively, of the *kdr* L1014F mutation (knockdown resistance genes from West Africa) [26]. Two types of farms growing cotton were selected for this study: these were in the villages of Dobao (11° 06 '38 9' 'N, 3° 00'35, 9 "W) and Kompla (11° 06 '6.0' 'N; 3° 00' 2 '' W). In the village of Dobao (a new biological cotton growing area since 2004), biological cotton was produced exclusively in all fields, while in Kompla (a conventional cotton area since 1960) both types of cotton were grown. The two villages are neighbors, with no physical barrier (swamps and forest) between them.

We also studied a third site, Soumousso (11° 01'46 "N 4° 02'45" W), where conventional cotton has been exclusively produced in continuation for at least ten years. This locality is situated 30 km from the city of Bobo-Dioulasso and 150 km from Dano. This study site was chosen as a positive, unbiased control to eliminate the possibility of contamination of breeding site water when conventional and biological cotton fields occur in the same village. This study was conducted from January to December 2013 with sample collections covering the rainy season from June to November 2013.

### Mosquito larva sampling and testing the toxicity of breeding site water from a conventional and biological cotton growing area

Mosquito larvae were collected during the rainy season (from June to November) corresponding to the period of insecticide treatments. The larvae of wild *An*. *gambiae* s.l. were collected in 10 breeding sites in the two farming sites. Small larvae stages (L1 and L2) were selected and used for biological tests with water taken from the breeding sites in the biological and conventional cotton fields. A susceptible strain of *An*. *gambiae* s.s. from Kisumu (from Kenya) was reared in the IRSS laboratory and used as a reference strain in this biological test.

The biological test is an indirect bioassay focused on the study of factors capable of inhibiting the normal growth of mosquito larvae in breeding sites. In our study, it was intended to show that in these larval waters there would be toxic compounds in water and sediment samples from breeding sites in the study sites which could affect the normal development of the larval stages of wild and susceptible *An*. *gambiae* s.l. populations. The protocol used in this evaluation is mainly based on comparison of the growth of larvae in test breeding sites (water and soil samples from agricultural areas under pesticide pressure) and in control breeding sites (water and soil samples from similar areas, but not under pesticide pressure). About 2,400 *An*. *gambiae* larvae were used in this test. 1200 larvae of each strain (wild and susceptible) were distributed into three treatment groups: one treatment group served as a negative control (using spring water free of any pesticide that is used in the IRSS insectary for the maintenance of colony mosquitoes) while the other treatment groups used water from the biological cotton breeding sites (Dobao) and from the conventional cotton breeding sites (Soumousso and Kompla). About 100 first and second stage wild *An*. *gambiae* s.l. larvae were exposed into 1L containers with breeding site water or spring water and reared to the adult stage. Each container was covered with mosquito netting and housed in a room where the relative humidity was between 70 to 80% and the temperature between 25–30°C. The larvae were washed and fed every two days. At emergence, adults were fed with glucose (5%). All emerging adults were counted to calculate the mortality rates per treatment group and older females (2 to 5 days post emergence) were used in tests of insecticide susceptibility and the efficacy of LLINs.

### Insecticide susceptibility assays

Adult susceptibility assays were carried out with two insecticides representative of those available for use in public health applications, using insecticide-treated filter papers at the diagnostic dose as recommended by the WHO [27] including one type II pyrethroid (0.05% deltamethrin), and one carbamate (0.1% bendiocarb). These molecules were selected because they are used directly or represent families of public health insecticides used for the impregnation of mosquito nets or in indoor residual spraying campaigns. Bioassays were conducted with 2 to 5 days old, non-blood fed adult female mosquitoes. Batches of 20–25 test mosquitoes were exposed for one hour to insecticide impregnated papers. Control mosquitoes (20–25 Kisumu strain females per test) were exposed for the same amount of time to untreated filter papers. After exposure, mosquitoes were transferred into insecticide-free observation tubes and maintained on 5% sucrose solution at a temperature ranging from 25 to 28°C. Final mortality in test and control mosquitoes was recorded 24 h after exposure. The threshold of susceptibility was fixed at 98% mortality rate for the two active molecules according to the WHO’s protocol [27]. When the mortality rates were between 98 and 80%, the population was considered as less susceptible while the population was considered resistant to the tested insecticides when mortality rates were below 80%.

### Efficacy tests of mosquito nets

In addition, the efficacy of nets was assessed using the WHO cone test. The nets tested were the PermaNet^®^ 3.0 and Interceptor^®^ which are nets used in the study area for the prevention of malaria. Susceptible and resistant *An*. *gambiae* s.s strains were tested separately. Batches of 10 unfed, 2–5 day old female *An*. *gambiae* s.s., including the standard susceptible Kisumu strain and wild insecticide-resistant adult mosquitoes, were placed inside the WHO cone and exposed simultaneously for three minutes before being transferred from cones to holding containers. The number of mosquitoes knocked down was then recorded 60 minutes after exposure and the final mortality was recorded 24 hours post-exposure. Survivors were maintained on 5% glucose solution. An untreated net was used as a negative control. Living and dead specimens from this test were separated and retained in silica gel for analysis of molecular forms and identification of resistance genes (*kdr* and a*ce-1*^*R*^).

### Species identification and genotyping (*kdr* and ace-*1*^*R*^)

Genomic DNA from mosquitoes was extracted with 2% cetyl trimethyl ammonium bromide (2% CTAB). Species and molecular forms of *An*. *gambiae* s.l. were identified and characterized, following previously published protocol of Santolamazza *et al*., [28]. Detection of mutations involved in insecticide resistance was also performed by PCR using the protocol of Martinez-Torres *et al*., [29] for the *kdr* L1014F mutation and of Weill *et al*., [30] for the ace-*1*^*R*^ G119S mutation.

### Chromatographic analysis of water and sediment samples from study sites

A part of each sample of breeding water was sent to a reference laboratory for identification and quantification of possible pesticide residues. The analysis by chromatographic methods aimed to determine the nature of these toxic compounds in water and sediments of breeding sites for *Anopheles gambiae* s.l. Two rounds of sampling were performed in this study. The first in late July / early August corresponding to the period before insecticide treatment and the second in late October / early November which corresponded to the period after treatment but before the cotton harvest. The quantity of samples required for pesticide residue analysis, were 1000 mL of water and 500 g of sediment / soil. Water and sediment samples from breeding sites were collected at random from different plots of the two comparative (biological and conventional) sites, around and within the cotton fields and following the vertical runoff of rainwater. Each identified plot was sampled in duplicate, mixed, and an amount of at least 1L was taken. Samples of breeding site water were collected using a dipper and put in beakers, whereas soil samples were collected using a soil auger. The breeding site water samples were placed in brown or clear glass bottles that were sterilized in advance and then wrapped with aluminum foil to protect the samples from light. These samples were then placed in coolers containing cold packs and delivered to the laboratory where they were analyzed within 24 hours. The pH value was checked in all samples (required value between 5 and 7.5). Storage of sediment samples (soil) was in plastic bags, which were kept away from moisture. All bottles and plastic bags were mark with an identifying code using permanent ink. All sampling points were geo-referenced. A total of twenty (20) water and sediment samples were randomly collected during the first and second sampling phase per site and sent to the reference laboratory for the detection and quantification of pesticide residue content.

Analytical methods aimed at identifying and quantifying pesticides in different compartments of the environment can be highly differentiated. The best methods are chromatographic methods. Chromatography can separate chemical compounds in a mixture of components by analyzing the mobile and stationary phases (contained in a column). When the mobile phase is a gas, gas chromatography separates the compounds according to their specific affinity for the stationary phase. When the mobile phase is a liquid (typically water-methanol mixtures, water-acetonitrile, etc.) liquid chromatography such as HPLC (High Performance Liquid Chromatography) is used. The pesticides were extracted from the samples of water and sediment (soil) successively by dichloro-methane and hexane. The extracts were then concentrated and purified. The compounds were identified and quantified by gas chromatography with the multi-residue analysis method [31] with a detection limit of 10 ppb. This is a method of detecting pesticides in water, soil and food, among other things. All the families of pesticides were tested according to their sensitivity to different detector chromatographs (GPC / HPLC).

### Ethics statement

The study did not involve vertebrates. Field studies did not involve endangered or protected species. For any locations/activities the authors state clearly that no specific permissions were required for these locations/activities. The permission has been obtained from local authorities of the town of Dano (Union of Cotton Producers of Dano, the traditional authorities of the two villages, the Chief Medical Officer of Dano) before the start of the study.

## Results

### Effects of toxic compounds present in breeding sites on development of *An*. *gambiae* larvae

**Fig 2:**
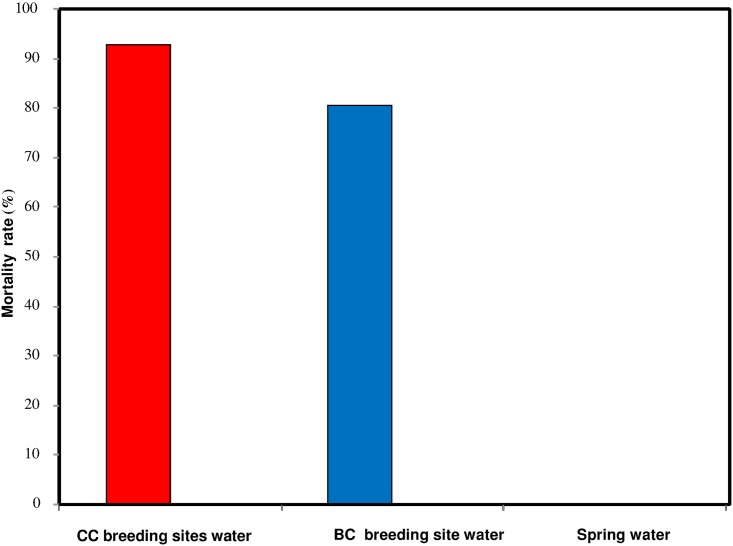
Rate of 1st instar *An*. *gambiae* Kisumu strain larvae reaching adult stages after exposure to breeding site waters from Soumousso (exclusive conventional cotton area), Dobao (exclusive biological cotton area), or Nasso (spring water control).

**Fig 3:**
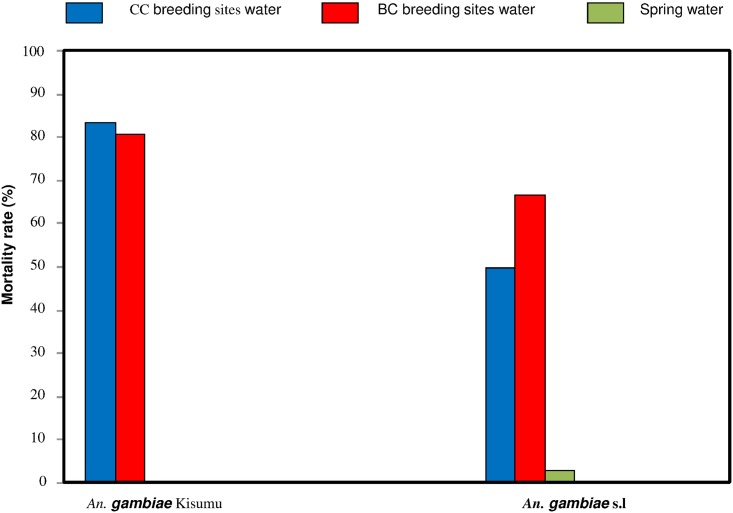
Rate of 1st instar *An*. *gambiae* s.l strain larvae reaching adult stages after exposure to breeding site waters from Kompla (conventional cotton area), Dobao (exclusive biological cotton area), or Nasso (spring water control).

The development of larvae was estimated by quantifying the proportion of first instar larvae reaching the adult stage. This is a good indicator to assess the toxicity of breeding site water in which larva may develop. This indicator takes into consideration the developmental cycle of larvae from first instars to adults. This cycle is relatively long and involves many interactions between larvae and constituents of breeding site waters which allows expressions of inhibiting factors in simulated breeding sites. The first results obtained with the susceptible Kisumu strain of *An*. *gambiae* exposed to the spring water from the region of Nasso and to breeding site water from a conventional cotton site (Soumousso) showed that with spring water (negative control), the larval mortality was very low (0.3%) whereas this mortality was very high (92.75%) in water from breeding sites in the conventional cotton area (Fig 2). The difference in mortality rates was highly significant (n = 400 P-Kisumu <0.0001, χ = 573, df = 1). Mortality rates obtained with spring water (negative control) were below 5% whatever the strain used, which confirms its non-contamination by insecticide residues or other pollutants. In addition, the mortality rate obtained with the susceptible strain exposed to breeding site waters from Dobao (80.75%), Kompla (83.5%) showed no significant difference (n = 400; P-Kisumu (Dobao *vs* Kompla) = 0.31, χ = 1.03, df = 1) (Fig 2). But, comparing the susceptible strain mortality in water collected at breeding sites from Soumousso (an exclusive conventional cotton area) and Dobao (an exclusive biological cotton area), we found a highly significant difference (n = 400, P-Kisumu (Dobao *vs* Soumousso) < 0.0001, χ = 30.83, df = 1) (Fig 2). Furthermore, mortality rates obtained with wild populations of *An*. *gambiae* s.l. exposed to breeding site waters from Dobao (49.75%) or Kompla (66.5%) and spring water (3%) were low compared to the mortality rates obtained with the susceptible strain (Fig 3). The statistical difference between the mortality rates obtained with wild populations was highly significant (n = 400, P-wild population (Dobao *vs* Kompla) < 0.0001; χ = 23.05, df = 1).

### Resistance to deltamethrin and reduced efficacy of Long Lasting Impregnated Nets (LLINs)

**Fig 4:**
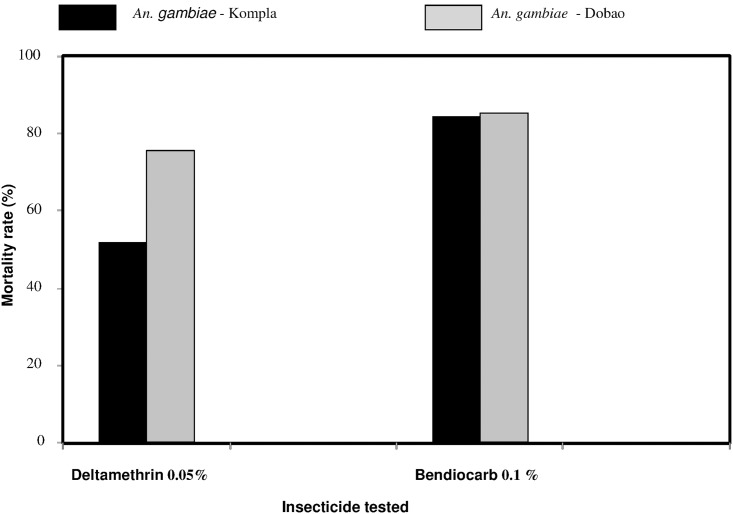
Mortality rates of *Anopheles gambiae* s.l. from two study sites observed after exposure to 0.05% deltamethrin and 0.1% bendiocarb.

Mortality rates varied according to the insecticide tested. The mortality observed with the susceptible Kisumu strain was 100% with both 0.05% deltamethrin and 0.1% bendiocarb. Mortalities observed with wild populations of *An*. *gambiae* s.l. in both localities showed intermediate resistance to bendiocarb, with 85.32% and 84.54%, respectively, from Dobao and Kompla, but the difference was not significant (P>0.05). Wild strains from both Dobao and Kompla were resistant to 0.05% deltamethrin with respective mortality rates of 75.96% and 52.04% (Dobao *vs*. Kompla, P = 0.48) (Fig 4).

Allele frequency of the *kdr* gene was not different between the two localities (0.95–0.98) and 0.4–0.33, respectively, for L1014F and L1014S (P> 0.05) and that of the ace-*1*^*R*^gene frequency did not vary between the two sites (0.12 for both sites).

**Fig 5:**
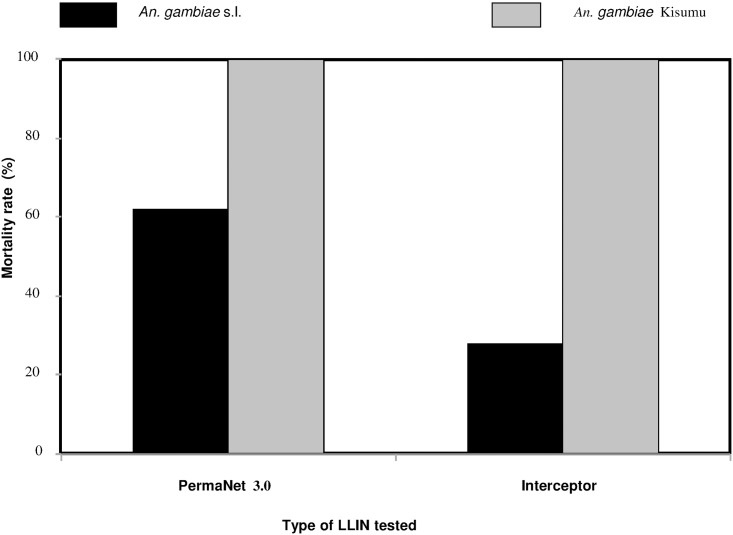
Efficacy of PermaNet^®^ 3.0 and Interceptor^®^ nets tested against susceptible and wild populations of *An*. *gambiae* s.l. study sites.

Furthermore, the results of the efficacy test performed on nets (PermaNet^®^ 3.0) used in the two study sites showed a low mortality rate of 62% with the local strain of *An*. *gambiae* s.l. (Fig 5). But this net caused 100% mortality in the susceptible reference Kisumu strain. The susceptible strain mortality was very low in experiments with the Interceptor^®^ net (28%).

### Pesticide residues detected in the water and sediment from larval breeding study sites using chromatographic analysis

**Fig 6:**
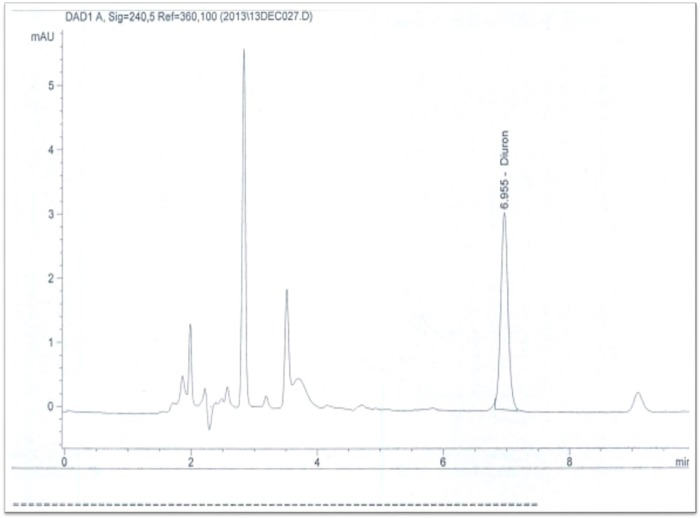
GC-μECD chromatogram of a sediment sample taken from a conventional cotton field (Kompla) before insecticide treatment showing the presence of diuron at a concentration of 105mm / kg soil.

**Fig 7:**
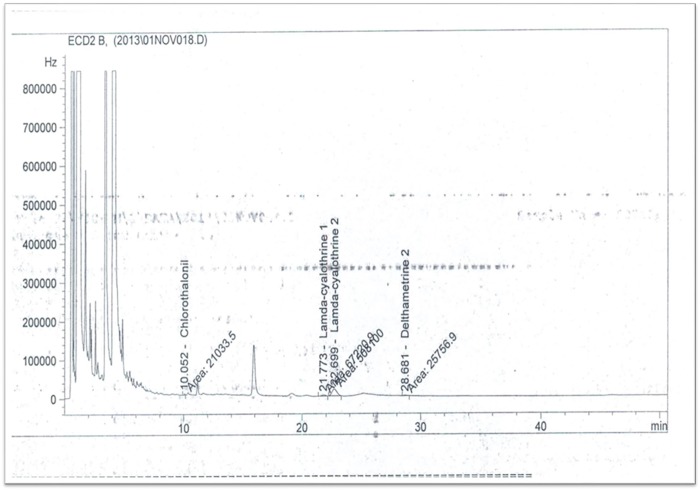
GC-μECD chromatograms of a water sample from a conventional cotton growing area after insecticide treatment of cotton fields showing the presence of deltamethrin and lambdacyhalothrin.

Chromatographic analysis of pesticide residues in the sediments of larval breeding sites located in Dobao and Kompla were performed in late July to early August (before treatment of fields with pesticides) showed the presence of diuron (organophosphate herbicide) at a concentration of 52.39 mg / kg of soil (Fig 6). This contamination was greater in the conventional cotton zone, where a maximum concentration of 105.5 mg diuron / kg of soil were found in Kompla (CC) compared to 29 mg diuron / kg of soil from Dobao (OC). In addition to diuron, other chemicals such as benzoyprop-ethyl, the fungicide chloroneb, pyridate, allethrin and bromacil were also identified in very low concentrations from soil residues in the collected samples. No pesticide residue was detected in any breeding site water at this earlier, pre-treatment collection time. But in late October through early November, corresponding to the period after treatment, the analysis revealed the presence of two pyrethroids in samples of breeding waters from the conventional cotton growing area. These pesticides were deltamethrin and lambda-cyhalothrin at concentrations, respectively, of 0.0164 μg / L and 1.49 μg / L (Fig 7).

## Discussion

The high mortality of the Kisumu reference strain larvae observed in the breeding site water from a conventional cotton growing area compared to spring water indicates the presence of toxic compounds in the water of the conventional cotton site. In our study, these compounds can be assumed to be pesticides, especially as the analysis of our water and soil samples revealed their presence in varying doses. Indeed, after treatments, these pesticides could be leached by rain water and could be found in the runoff from the fields that constitute the water found in many breeding sites. The insecticide residues in these deposits would exert a selection pressure on larval mosquito populations that are there. The analysis of the toxicity test results performed with wild populations of *An*. *gambiae* s.l. from the two study sites showed a statistically significant difference. These results are consistent with our initial hypothesis that the presence of pesticide residues in breeding sites around conventional cotton cultivation plots could be the basis for the selection and spread of insecticide-resistant *An*. *gambiae* sl. The failure to observe a significant difference in allele frequencies of the *kdr* gene in mosquitoes collected from the two different locations may be explained by the lack of a physical barrier between the conventional and organic cotton fields. Indeed, the distance between some fields of organic and conventional growing areas is not large, with an average of one kilometer in some localities where both types of fields overlap. Therefore runoff rainwater from conventional cotton growing areas containing pesticide residues could easily fill breeding sites around organic cotton fields that have not received pesticide treatment. Moreover, the mortality rates in mosquito bioassays and the resistance status of wild *An*. *gambiae* s.l. are similar between these two areas. Indeed, the results of WHO tube tests confirmed intermediate resistance to bendiocarb and high resistance to deltamethrin in the two sites that were previously described by Dabire *et al*., [12], which also showed expanded vector resistance to carbamates and organophosphates in most cotton growing areas in Burkina Faso. These results reinforce those of our bioassays and the data suggest that vector control tools (LLINs) based on pyrethroids in the two study sites are likely to be very compromised. The resistance observed with deltamethrin may be primarily due to the intensive use of this insecticide [13, 19] for the treatment of cotton fields. However, the use of LLINs, especially during the free distribution of insecticide-treated nets in 2010 and 2013, could be a second source of resistance selection pressure because most of these LLINs are impregnated with deltamethrin. Furthermore, the results of tests with commercial nets are in agreement with the results of our mosquito bioassays as evidenced by the level of resistance of *An*. *gambiae* s.l. in these communities and the high frequency of the *kdr* mutation L1014F. These results clearly confirm the existence of selection pressure for resistance exerted by the intensive use of insecticides in agriculture on larval populations of *An*. *gambiae* s.l. in agricultural areas in general, and with cotton farming in particular.

Two insecticides have been found in the water in the conventional cotton breeding zones of Kompla. Deltamethrin and lambdacyhalothrin (S1 Table) were found in samples collected during the month of October, 2013, while those collected from late July through early August showed no traces of pesticides in the samples analyzed. The detection of insecticide molecules in this time period reflects the treatments carried out for crop protection from insect pests between September and October following the phenology of cotton plants and the cotton treatment window adopted by producers. The identification of pesticide residues in water and soil collections reinforces all the studies that have suggested a role of agriculture in the selection of resistance of malaria vectors to insecticides [19, 32] and especially in cotton growing areas [13, 19]. In view of these results, we can conclude that the intense use of insecticides in conventional cotton growing areas is highly toxic to the susceptible Kisumu strain of *An*. *gambiae*. Even bioassays using insecticide-impregnated papers have shown similar results with significantly higher mortality in mosquitoes collected from the organic cotton growing area. The large-scale promotion of this agriculture that is free from chemical insecticides was probably an advantage that allowed for the presence of insecticide-sensitive of malaria vectors in these localities. We can conclude from this study that it is necessary, more than ever, to undertake farming practices that use little or no chemical insecticides (organic horticulture), which is expected to restore long-term insecticide-sensitive populations of *Anopheles gambiae* s.l. and which will contribute to the effectiveness of our control tools based on pyrethroids (LLINs and IRS) in these areas of high insecticide use.

## Supporting information

S1 Table Chromatographic analysis result of a water sample from a conventional cotton growing site. (PDF)

S2 Table Chromatographic analysis result of a sediment sample from a conventional cotton growing site. (PDF)
