# Supplementary material for: Evidence that agricultural use of pesticides selects pyrethroid resistance within Anopheles gambiae s.l. populations from cotton growing areas in Burkina Faso, West Africa
Source: PLoS One. 2017 Mar 2;12(3):e0173098. doi: 10.1371/journal.pone.0173098 (PMC5333875; doi:10.1371/journal.pone.0173098)
Supplement: S1 Table — (PDF) [file pone.0173098.s001.pdf]

# RAPPORT D'ANALYSE N°

**DEPARTEMENT CHARGE DU CONTRÔLE :**
**Direction de la Toxicologie, du Contrôle**
**de l'Environnement et de l'hygiène Publique**
**------------------------------**
Service des Contaminants, de Pesticides et des Engrais

| | |
|---|---|
| Codes clients | : **00751/001-00752/001-00753/001-00754/001-00755/001-00762/001- 00763/001-00764/001-00765/001** |
| Date du prélèvement | : **16-10-2013** |
| Lieu du prélèvement | : **Compla 1, Compla 2 et Dayere** |
| Date de réception | : **12-08-2013** |
| But des analyses | : **Recherche de résidus de pesticides** |
| Période d'analyse | : **Du 29 octobre 17 décembre 2013** |
| Identité du préleveur | : **Client** |
| Nature des échantillons | : **Eau** |

| Pesticides Recherchés | Méthodes | Désignation et résultats | | | | | | | | | | Valeurs guides ** (µg/l) |
|---|---|---|---|---|---|---|---|---|---|---|---|---|
| | | 00751/001 CONV 5 / Compla 2 | 00752/001 CONV 6 / Compla 2 | 00753/001 BIO 1 / Compla 1 | 00754/001 BIO 2 / Compla 1 | 00755/001 BIO. 3 / Dayere | 00762/001 CONV. 1 / Compla 2 | 00763/001 CONV. 2 / Compla 2 | 00764/001 CONV. 3 / Compla 2 | 00765/001 CONV. 4 / Compla 2 | 00761/001 CONV. 6 / Dayere | |
| **PYRETHRINOIDE DE SYNTHESE** | | | | | | | | | | | | |
| Cypermethrine | | < LD* | < LD* | < LD* | < LD* | < LD* | < LD* | < LD* | < LD* | < LD* | < LD* | - |
| Deltamethrine | | < LD* | < LD* | < LD* | < LD* | < LD* | < LD* | < LD* | < LD* | < LD* | **0,0147** | - |
| Lamda Cyhalothrine | | < LD* | < LD* | < LD* | < LD* | < LD* | < LD* | < LD* | < LD* | < LD* | **1,49** | - |
| Permethrine | | < LD* | < LD* | < LD* | < LD* | < LD* | < LD* | < LD* | < LD* | < LD* | < LD* | - |
| Tetramethrine | | < LD* | < LD* | < LD* | < LD* | < LD* | < LD* | < LD* | < LD* | < LD* | < LD* | - |
| **ORGANOPHOSPHORES / COMPOSES AZOTES :** | Méthode Multi résidus d'origine DFG*** | | | | | | | | | | | |
| Chlorpyrifos ethyl | | < LD* | < LD* | < LD* | < LD* | < LD* | < LD* | < LD* | < LD* | < LD* | < LD* | - |
| Chlorpyrifos methyl | | < LD* | < LD* | < LD* | < LD* | < LD* | < LD* | < LD* | < LD* | < LD* | < LD* | - |
| Diazinon | | < LD* | < LD* | < LD* | < LD* | < LD* | < LD* | < LD* | < LD* | < LD* | < LD* | - |
| Dichlorvos | | < LD* | < LD* | < LD* | < LD* | < LD* | < LD* | < LD* | < LD* | < LD* | < LD* | - |
| Dimethoate | | < LD* | < LD* | < LD* | < LD* | < LD* | < LD* | < LD* | < LD* | < LD* | < LD* | - |
| Fenitrothion | | < LD* | < LD* | < LD* | < LD* | < LD* | < LD* | < LD* | < LD* | < LD* | < LD* | - |
| Fipronil | | < LD* | < LD* | < LD* | < LD* | < LD* | < LD* | < LD* | < LD* | < LD* | < LD* | - |
| Malathion | | < LD* | < LD* | < LD* | < LD* | < LD* | < LD* | < LD* | < LD* | < LD* | < LD* | - |
| Methidathion | | < LD* | < LD* | < LD* | < LD* | < LD* | < LD* | < LD* | < LD* | < LD* | < LD* | - |
| Omethoate | | < LD* | < LD* | < LD* | < LD* | < LD* | < LD* | < LD* | < LD* | < LD* | < LD* | - |
| Parathion ethyl | | < LD* | < LD* | < LD* | < LD* | < LD* | < LD* | < LD* | < LD* | < LD* | < LD* | - |
| Pyridaphenthion | | < LD* | < LD* | < LD* | < LD* | < LD* | < LD* | < LD* | < LD* | < LD* | < LD* | - |
| Pyrimiphos methyl | | < LD* | < LD* | < LD* | < LD* | < LD* | < LD* | < LD* | < LD* | < LD* | < LD* | |
| **CARBAMATES et AUTRES:** | | | | | | | | | | | | |
| Imazalil | | < LD* | < LD* | < LD* | < LD* | < LD* | < LD* | < LD* | < LD* | < LD* | < LD* | - |
| Quintozene | | < LD* | < LD* | < LD* | < LD* | < LD* | < LD* | < LD* | < LD* | < LD* | < LD* | - |

(*) : Limite de Détection
(***) : DFG : Deutsche Forschungsgemeinschaft

***<u>Avertissement</u> : Ce document ne doit pas être reproduit sans autorisation écrite du LNSP.***
***Ce rapport d'analyse n'est valable que pour l'échantillon analysé.***

**Le Chef de service des Contaminants, des Pesticides et des Engrais**

**Le Directeur de la Toxicologie du Contrôle de l'Environnement et de l'Hygiène Publique P/O**

***Avertissement* : Ce document ne doit pas être reproduit sans autorisation écrite du LNSP.**
***Ce rapport d'analyse n'est valable que pour l'échantillon analysé.***
