# Supplementary material for: Evidence that agricultural use of pesticides selects pyrethroid resistance within Anopheles gambiae s.l. populations from cotton growing areas in Burkina Faso, West Africa
Source: PLoS One. 2017 Mar 2;12(3):e0173098. doi: 10.1371/journal.pone.0173098 (PMC5333875; doi:10.1371/journal.pone.0173098)
Supplement: S2 Table — (PDF) [file pone.0173098.s002.pdf]

RAPPORT D'ANALYSE N°

DEPARTEMENT CHARGE DU CONTRÔLE :
Direction de la Toxicologie, du Contrôle
de l'Environnement et de l'hygiène Publique

Service des Contaminants, de Pesticides et des Engrais

Codes clients	: **00746/001-00747/001-00748/001-00749/001-00750/001 00756/001-00757/001-00758/001-00759/001-00760/001-00761/001**
Date du prélèvement	: **16-10-2013**
Lieu du prélèvement	: **Compla 1, Compla 2 et Dayere**
Date de réception	: **12-08-2013**
But des analyses	: **Recherche de résidus de pesticides**
Période d'analyse	: **Du 29 octobre 17 décembre 2013**
Identité du préleveur	: **LNSP**
Nature des échantillons	: **Sol**

***Avertissement* : Ce document ne doit pas être reproduit sans autorisation écrite du LNSP.**
Ce rapport d'analyse n'est valable que pour l'échantillon analysé.

Pesticides Recherchés	Méthodes	Désignation et résultats											Valeurs guides ** (µg/l)
		00746/001 BIO. 1 / Compla 1	00747/001 BIO. 2 / Compla 1	00748/001 BIO. 3 / Compla 1	00749/001 BIO. 4 / Compla 1	00750/001 BIO. 5 / Dayere	00756/001 CONV. 1 / Compla 2	00757/001 CONV. 2 / Compla 2	00758/001 CONV.3 / Compla 2	00759/001 CONV. 4 / Compla 2	00760/001 CONV. 5 / Compla 2	00761/001 CONV. 6 / Dayere	
**PYRETHRINOIDE DE SYNTHESE**	Méthode Multi résidus d'origine DFG***												
Cypermethrine		< LD*	< LD*	< LD*	< LD*	< LD*	< LD*	< LD*	< LD*	< LD*	< LD*	< LD*	-
Deltamethrine		< LD*	< LD*	< LD*	< LD*	< LD*	< LD*	< LD*	< LD*	< LD*	< LD*	< LD*	-
Lamda Cyhalothrine		< LD*	< LD*	< LD*	< LD*	< LD*	< LD*	< LD*	< LD*	< LD*	< LD*	< LD*	-
Permethrine		< LD*	< LD*	< LD*	< LD*	< LD*	< LD*	< LD*	< LD*	< LD*	< LD*	< LD*	-
Tetramethrine		< LD*	< LD*	< LD*	< LD*	< LD*	< LD*	< LD*	< LD*	< LD*	< LD*	< LD*	-
**ORGANOPHOSPHORES / COMPOSES AZOTES :**													
Chlorpyrifos ethyl		< LD*	< LD*	< LD*	< LD*	< LD*	< LD*	< LD*	< LD*	< LD*	< LD*	< LD*	-
Chlorpyrifos methyl		< LD*	< LD*	< LD*	< LD*	< LD*	< LD*	< LD*	< LD*	< LD*	< LD*	< LD*	-
Diazinon		< LD*	< LD*	< LD*	< LD*	< LD*	< LD*	< LD*	< LD*	< LD*	< LD*	< LD*	-
Dichlorvos		< LD*	< LD*	< LD*	< LD*	< LD*	< LD*	< LD*	< LD*	< LD*	< LD*	< LD*	-
Dimethoate		< LD*	< LD*	< LD*	< LD*	< LD*	< LD*	< LD*	< LD*	< LD*	< LD*	< LD*	-
Diuron		< LD*	< LD*	**29,03**	< LD*	< LD*	< LD*	**22,63**	**105.50**	< LD*	< LD*	< LD*	-
Glyphosate		< LD*	< LD*	< LD*	< LD*	< LD*	< LD*	< LD*	< LD*	< LD*	< LD*	< LD*	-
Fenitrothion		< LD*	< LD*	< LD*	< LD*	< LD*	< LD*	< LD*	< LD*	< LD*	< LD*	< LD*	-
Fipronil		< LD*	< LD*	< LD*	< LD*	< LD*	< LD*	< LD*	< LD*	< LD*	< LD*	< LD*	-
Malathion		< LD*	< LD*	< LD*	< LD*	< LD*	< LD*	< LD*	< LD*	< LD*	< LD*	< LD*	-
Methidathion		< LD*	< LD*	< LD*	< LD*	< LD*	< LD*	< LD*	< LD*	< LD*	< LD*	< LD*	-
Omethoate		< LD*	< LD*	< LD*	< LD*	< LD*	< LD*	< LD*	< LD*	< LD*	< LD*	< LD*	-
Parathion ethyl		< LD*	< LD*	< LD*	< LD*	< LD*	< LD*	< LD*	< LD*	< LD*	< LD*	< LD*	-
Pyridaphenthion		< LD*	< LD*	< LD*	< LD*	< LD*	< LD*	< LD*	< LD*	< LD*	< LD*	< LD*	-
Pyrimiphos methyl		< LD*	< LD*	< LD*	< LD*	< LD*	< LD*	< LD*	< LD*	< LD*	< LD*	< LD*	-
**CARBAMATES et AUTRES:**													
Imazalil		< LD*	< LD*	< LD*	< LD*	< LD*	< LD*	< LD*	< LD*	< LD*	< LD*	< LD*	-
Quintozene		< LD*	< LD*	< LD*	< LD*	< LD*	< LD*	< LD*	< LD*	< LD*	< LD*	< LD*	-

(*) : Limite de Détection
(***) : DFG : Deutsche Forschungsgemeinschaft

***Avertissement* : Ce document ne doit pas être reproduit sans autorisation écrite du LNSP.**
Ce rapport d'analyse n'est valable que pour l'échantillon analysé.
007466-00747-00748-00749-00750-00756-00757-00758-00759-00760-00761 Echantillons de sol

Remarque

Les molécules de pesticides (Alléthrine Benzoylprop-ethyl, Bromacil, Chloroneb, Dioxacarb, Pyridate) ont été retrouvées dans certains échantillons (voir le tableau ci-dessous),

Molécule des pesticides	Désignation et résultats									
	00746/001 BIO. 1 / Compla 1	00747/001 BIO. 2 / Compla 1	00749/001 BIO. 4 / Compla 1	00750/001 BIO. 5 / Dayere	00756/001 CONV. 1 / Compla 2	00757/001 CONV. 2 / Compla 2	00758/001 CONV.3 / Compla 2	00759/001 CONV. 4 / Compla 2	00760/001 CONV. 5 / Compla 2	00761/001 CONV. 6 / Dayere
Alléthrine		+								
Benzoylprop-ethyl							+			
Bromacil					+					
Chloroneb	+	+	+	+			+	+	+	
Dioxacarb				+						
Pyridate					+					

Le Chef de service des Contaminants, des Pesticides et des Engrais

Le Directeur de la Toxicologie du Contrôle de l'Environnement et de l'Hygiène Publique P/O

Abdoulaye SAKO

Mme Bernadette P. SOURABIE

***Avertissement* : Ce document ne doit pas être reproduit sans autorisation écrite du LNSP.**
Ce rapport d'analyse n'est valable que pour l'échantillon analysé.
007466-00747-00748-00749-00750-00756-00757-00758-00759-00760-00761 Echantillons de sol
